# Characteristics of Occupational Injuries among Spanish Nursing Workers

**DOI:** 10.3390/healthcare10020220

**Published:** 2022-01-24

**Authors:** María del Carmen Rey-Merchán, Antonio López-Arquillos, Ana María Rey-Merchán

**Affiliations:** 1 Consejería de Educación y Deporte, Junta de Andalucía, 29071 Málaga, Spain; mmccrrmm@gmail.com; 2 Departamento de Economía y Administración de Empresas, Universidad de Málaga, 29071 Málaga, Spain; 3 Consejería de Salud, Servicio Andaluz de Salud, Andalucía, 29400 Ronda, Spain; anreme2@hotmail.com

**Keywords:** nursing, occupational accidents, safety risk, workplace

## Abstract

Introduction: Nursing professionals face a multitude of daily occupational hazards that can cause occupational accidents. AIM: The objective of this work is to analyze the personal variables included in official accident reports, to evaluate their influence on occupational accidents suffered by nursing and nursing assistant professionals. Methodology: A total of 187,821 occupational accidents recorded in Spain from 2011 to 2019 were analyzed in the sector using contingency tables, chi-square, and corrected standardized residuals. Results: The results showed that the older the professional, the probability that once the accident had occurred, its severity would be more serious. Regarding gender, men are more likely to suffer more serious accidents compared to accidents registered by women. Results about the length of service and nationality did not reach statistical significance in the group of accidents analyzed. Conclusions: The planning of preventive measures must be adapted to the profiles of the workers in the most vulnerable sector.

## 1. Introduction

The working conditions related to nursing and nursing assistant positions often entail a series of common risks that can affect the health of workers if adequate preventive measures are not considered. There are many existing studies on the prevalence of diseases and occupational accidents that focus on the group of nurses and/or nursing assistants [1,2,3,4,5]. These cited studies report that occupational accidents are mainly associated with musculoskeletal disorders, falls, blows with objects, and cuts caused by sharp-pointed materials, frequently contaminated by body fluids of patients, or with an inadequate workload distribution. An example of this is found in studies such as the one carried out in hospitals in Brazil, where 34.7% of the accidents that occurred among nurses were associated with musculoskeletal problems [6]. In countries such as Chile, it was concluded that blows to different parts of the body, as well as falls caused by slipping, are the most common accidents, followed by injuries caused by sharp-pointed clinical material [7]. In Spain, the problem of occupational accidents among the professionals of the group is similar [8,9]. Although there are many records on occupational accidents of nurses and nursing assistants, it has been detected that only the most serious are reported: There are still around 80% of workers who do not report musculoskeletal problems [10]; therefore, working on effective measures to avoid them becomes complicated. Due to the sick leave that follows this type of accident, it is relatively frequent to find studies on the type of accidents that lead to financial compensation and long-term sick leave [11].

The majority of previous research is based on occupational questionnaires and reported injuries. In the first group, different occupational questionnaires were used. Some authors used Epworth Sleepiness Scale and the Workload Scale and found that nurses had very high rates of occupational accidents and that the heavy work conditions affected occupational accidents [12]. Similarly, research based on the survey of shiftwork, sleep, and health among Norwegian nurses identified that quick returns between consecutive shifts was associated with a higher risk of occupational accidents [13]. Aligned with that, and using three questionnaires (organizational, occupational injuries checklist, and the NASA-Task Load Index (TLX)), it was pointed out that working in rotating shifts and work overload was significantly related to all injuries [14]. Other authors designed and distributed a specific safety questionnaire for nurses. They found that the training of nurses was necessary for improving both safety compliance and safety participation, and thereby reducing occupational accidents [15].

In the second group, the number of studies based on reported injuries is low. For instance, the research conducted in a large Belgian university hospital concluded that female and older employees are more vulnerable to accidents [16].

Literature found based on questionnaires and accident reports was frequently based only on a local sample of workers from a hospital. This limitation is an important barrier to generalizing the results obtained. Based on all of the above, it is necessary to understand the influence of the different personal factors present in accidents, to avoid the great variety of biological, physical, and chemical hazards during the performance of their functions and based on these variables improve the preventive measures in the sector.

Therefore, the objective of this work is to analyze the personal variables included in official accident reports, to evaluate their influence on occupational accidents suffered by nursing and nursing assistant professionals.

## 2. Methodology

In Spain, the notification of work accidents is mandatory by law [17], which is why the competent labor authority registers all accidents officially notified by the electronic procedure. Official accident reports in Spain include 58 variables such as gender, age of the workers, day of the week, severity, or place of the accident, among others. Then, accidents can be classified according to all variables included in the official report. Frequently, accidents are grouped according to the National Classification of Economic Activities or CNAE, which allows the classification and grouping of production units according to the activity they carry out. Another important variable is the National Classification of Occupations (CNO), which is the Spanish national adaptation of the International Standard Classification of Occupations. In this study, we have worked with data on nursing (CNO code = 212) and nursing assistant activities (CNO code = 561). Cited codes include the following occupations: non-specialized nurses, specialized nurses, midwives, hospital nursing assistants, primary care nursing assistants, healthcare workers to people in health services not classified above, and home personal care workers.

**Figure 1:**
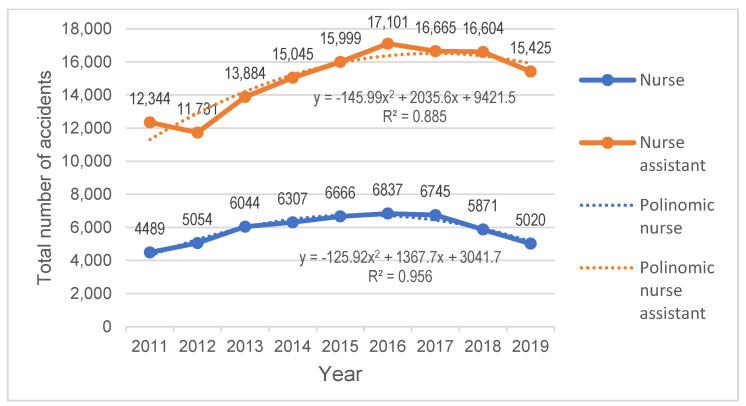
Distribution of occupational accidents by year.

**Figure 2:**
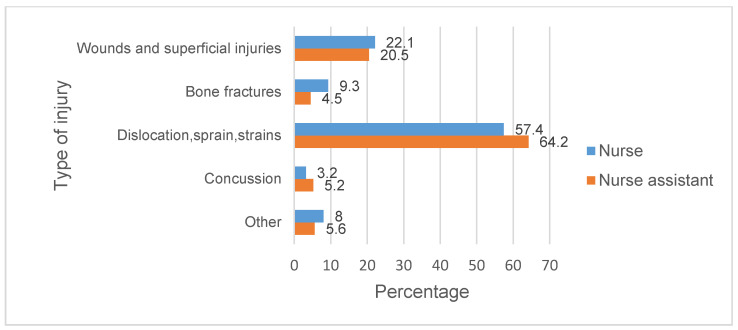
Distribution of the accidents based on the type of injury.

When extracting data on accidents related to our groups, a total of 187,821 cases were obtained between the years 2011 and 2019. A cross-sectional study was carried out based on cited accidents. A total of 53,033 cases correspond to work-related accidents suffered by the group of nurses, while the rest correspond to the group of auxiliaries of nursing. It can be observed (Figure 1) that occupational accidents increased from 2011 until 2016, and then they decreased until 2019. In contrast, the total number of nurses employed in Spain increased continuously every year from 2011 to 2019 [18]. Then, the reduction of the number of accidents was not motivated by a decrease in employed professionals. In addition, the distribution of the accidents according to the injury suffered by the workers is shown in Figure 2.

Once the cases had been identified, a frequency analysis of the personal variables for our study was carried out and contingency tables were made that relate each of these variables to the severity of the accident (light, serious, very serious, or fatal). Personal variables selected to describe the personal profile of the worker were age, gender, length of service, and nationality.

When performing the contingency tables, the program calculated the chi-square relationship and the corrected standardized residuals. The chi-square relationship or Pearson distribution determined whether there was a relationship between two categorical variables. If the value obtained was higher than a certain critical value, the independence hypothesis was rejected and it was established that there was a relationship between both variables. Finally, ratios were calculated to represent the percentage of occurrence of each level of severity. The LAR (Light Accident Ratio) calculates the percentage of minor accidents of each value or range of values of a variable to the total of minor accidents, the SAR (Serious Accident Ratio) calculates the proportion of serious accidents, VSAR (Very Serious Accident Ratio) calculates the proportion of very serious accidents, FAR (Fatal Accident Ratio) calculates the proportion of fatal accidents and, finally, the TAR index (Total Accident Ratio) represents the percentage of accidents of each value or range of values of a variable to the total number of registered accidents. For the statistical analysis, the computer program IBM SPSS Statistics 23 (IBM, Armonk, NY, USA) was used. The methodology described has already been used successfully in a similar way in previous studies [19,20].

Additionally, odds ratio values based on the logistic regression model were obtained for dichotomic variables studied [21]. The strength of the relationship between variables analyzed was calculated using an adjusted odds ratio with a confidence interval of 95%.

**Figure 3:**
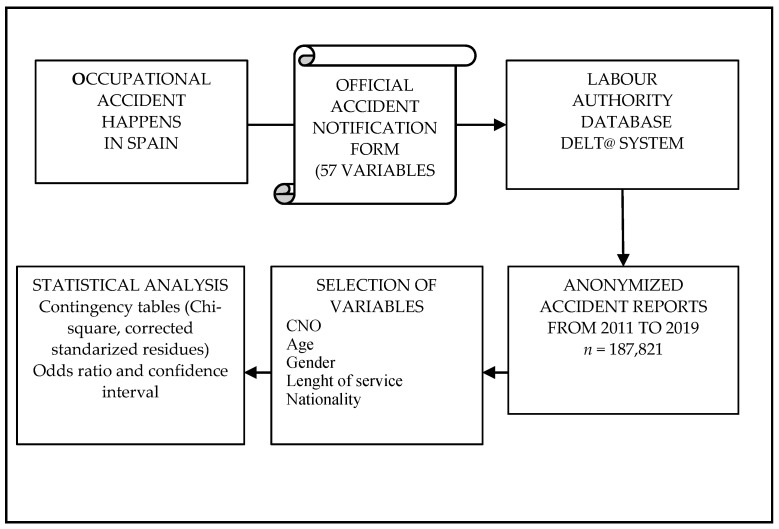
Methodology flowchart.

A summary of the methodology carried out is shown in Figure 3.

## 3. Results and Discussion

The results of the main personal variables studied are described below.

### 3.1. Age

In this section, the variables degree of injury and age were related. The chi-square value obtained was 203 for nurses and 193 for nursing assistants. The asymptotic significance obtained a value less than 0.05, so it could be concluded that there is a dependence between the severity of the injury and the age of the worker. In most of the cases, the residual adjustment obtained is greater than 1.96, thus corroborating the dependence between the variables. In the following Table 1, the values whose residual value obtained an absolute value greater than 1.96 have been marked with *.

It was observed that the majority of accidents are minor and occur more frequently among health workers between 50 and 65 years old, closely followed by workers between 40 and 49 years old. On the other hand, it is observed that it is in the age group of 50–65 years that accidents considered as serious and very serious are concentrated, assuming of all accidents of this severity. It could be said that the interval 50–65 is the one with the most accidents regardless of the severity, followed by the 40–49 segment. On the other hand, from this Table, it can be inferred that workers younger than 40 years old are the least likely to suffer accidents classified as serious, very serious, or fatal.

Additionally, workers were grouped into young and senior workers. The odds ratio obtained (Table 2) pointed out that accidents suffered by young workers are more probably to be light than accidents suffered by senior nurses and nurse assistants.

In the field of nursing, the number of publications that study the prevalence of occupational accidents of all kinds and the age of the workers who suffer them is low. It is remarkable that the official report published by the Bureau of Labor Statistics in 2018 [22] showed a similar distribution of occupational accidents by age among nurses. However, it is common to find studies in which the age variable has been introduced in the analysis, relating it to the prevalence of certain accidents or a particular risk. Thus, Heiden [23] studied the relationship between age and musculoskeletal type work injuries and concluded that there were significant differences in the frequency with which they occurred when personnel made up of young and middle-aged workers were compared with older personnel, the former being more prone to accidents and the latter being more serious. Similarly, it was reported that physical capabilities might differ from the young to the old age nurses and therefore the occurrence of injuries shows an important increase [24]. In contrast, other authors did not find a correlation between occupational musculoskeletal injuries and age [25]. Finally, preventive measures adapted to age were recommended. In contrast, other authors [26] determined that older workers experience fewer musculoskeletal injuries than younger workers due to greater skill and experience in the tasks they perform, but that when injuries do occur, they are more severe so recovery time is longer. Regarding the incidence of injuries by sharpshoot material [27], these injuries were found to occur more frequently among young nurses. These findings seem to indicate that in an accident, the relationship between the worker’s age and the frequency and severity of the injury produced is strongly influenced by the type of risk that the accident causes.

### 3.2. Gender

Regarding the gender of the worker, the results obtained are shown in Table 3. Although the percentage of women injured is higher, this percentage is motivated by a greater presence of women in the sector. This was shown by the statistics published by the INE in 2013, in which it was revealed that 84.4% of the workers of the Spanish nurses and nursing auxiliaries are made up of women. However, the percentage of injured women is higher than the percentage of occupied women among nurses (87.5%) and nursing assistants (92.2%).

It should be noted that in the case of men, the percentage of accidents increases as the severity of accidents increases, growing from 12.3% nurses in minor accidents to 32% in the case of fatal accidents. These results are aligned with the odds ratio calculated based on the sex of the worker. The more severe the accident is, the more probable it is that the accident was suffered by a man (Table 4). Aligned with these results, other authors found a statistically significant association with gender and light accidents with percutaneous injuries. They pointed out that women presented almost twice the chance of suffering percutaneous accidents when compared to men [28].

There are several reasons studied in other sectors that can motivate this difference in terms of the accident rate between men and women, such as the workload, the different perception of risk, or the percentage of occupational traffic accidents among the total fatal accidents [29,30,31]. Gender differences in other occupational issues such as bullying exposure and burnout also need to be addressed [32].

### 3.3. Length of Service

Regarding the worker’s seniority in the company, no significant differences were found between nurses and nurse assistants. In the majority of odds ratio calculated (Table 5), the confidence interval (C.I) contents the unit, then the results obtained were not statistically significant.

Considering the two groups together, it was obtained that for the first 5 years, 25.4% of the accidents, almost half of those that occurred in those first few years, occurred during the first 12 months of seniority. The second year already only accumulates 9.4% of the total accidents and, progressively, the fifth year is reached with only 5.7% of the incidents. In the USA, other authors found that about half of the injured nurses were long-term employees who worked for their employer for 5 or more years [22]. Although the variable under study here refers to the length of service in the company, in reality, it is also intrinsically linked to experience. In this sense, the results obtained are in line with some studies published about the experience. Thus, Delloiacono [26] concluded that personnel with more experience report fewer injuries of musculoskeletal origin compared to professionals with less seniority. Similarly, other authors demonstrated that the lack of skill in the handling of the instruments due to a lack of experience can be a variable that notably influences injuries due to sharpshoot material [33]. In contrast, other authors concluded that reported musculoskeletal disorders were significantly correlated with years of experience, nurse-to-patient ratios, and chronic occupational fatigue [34]. Aligned with these results, it was pointed out that the years of employment of nurses have implications for the incidence of musculoskeletal symptoms at the workplace [35].

### 3.4. Nationality

Regarding the professional’s nationality, no statistically significant differences were found between the worker’s country of origin and the severity of the injuries, once the accident occurred. Contingency tables obtained low values for chi-square and corrected standardized residues. Similarly, the odds ratio did not reach statistical significance.

In previous literature, it was found that the prevalence of work-related musculoskeletal symptoms was significantly lower in foreign-educated nurses than in national-educated nurses, but the difference was not significant in the multivariable analyses [36]. This fact contrasts with other sectors such as construction or agriculture where foreign workers are more likely to suffer more serious accidents [37,38]. One of the reasons may be that nursing professionals have similar training in occupational risk prevention, regardless of their country of origin. In the same way, performing similar tasks regardless of what country in the world they work in, nursing professionals adapt to their jobs faster than in other professions. Another reason for the absence of nationality significance is the low percentage of foreign workers (>2%) in the accidents data analyzed.

However, it is important to note that in results from previous research [39], migrant nurses of different origins showed differences in their physiological responses to stress, and this might be a cause of impaired health among migrant and native nurses.

## 4. Conclusions

Considering the results, it can be concluded that some personal variables influence the severity of occupational accidents in nurse and nursing assistant professionals. In this sense, it was found that the older the worker, the probability that once the accident has occurred, the greater the severity of the accident. Regarding sex, men have a greater probability that their accidents are more serious compared to the accidents registered by women. Regarding the nationality of the worker, it was not found to be a variable that influenced the severity of the accident.

Personal variables such as age and gender should be considered in the mitigation actions in future safety planning. The results obtained serve as a starting point for the design of specific preventive plans adapted to the profiles of workers in the sector most vulnerable to more serious accidents. They can be useful in devising strategies for reducing the high rates of injury and illness in nurses and nursing assistants.

Despite this, in Spain it is compulsory to report all occupational accidents to the Labor Authorities; however, it is possible that some accidents happened, but they were not notified. Therefore, a possible bias caused by under-reported accidents might be considered.

Further in-depth research is necessary to determine the influence of other organizational factors (size of the company, safety management, workload) and temporal factors (hour of the accident, day of the week).

Finally, the combination of safety surveillance with accidents reports data in future research could improve the current results about occupational accidents among nurses and nursing assistants.

## Figures and Tables

**Table 1:** Contingency table Age vs. Severity.

Severity	Light				Serious				Very Serious				Fatal				Total	
Category	Nurse		N.Assistants		Nurse		N.Assistants		Nurse		N.Assistants		Nurse		N.Assistants		Nurse TAR	N.Assistants FAR
Age	Num	LAR	Num	LAR	Num	SAR	Num	SAR	Num	VSAR	Num	VSAR	Num	FAR	Num	FAR		
18–29	5924	11.3	18,497	13.8	27	4.1	22	4.4	4 *	12.1	1 *	5.9	1 *	6.7	1 *	4.0	11.2	13.7
30–39	12,805	24.5	26,925	20.1	70	10.6	49	9.8	4 *	12.1	2 *	11.8	4 *	26.7	2 *	8.0	24.3	20.0
40–49	12,298	23.5	36,414	27.1	126	19.0	110	22.0	5 *	15.2	3 *	17.6	1 *	6.7	14	56.0	23.4	27.1
50–65	21,244	40.6	52,255	38.9	439	66.2	320	63.9	20	60.6	11	64.7	9 *	60.0	7 *	28.0	40.9	39.0
No data	51 *	0.1	154 *	0.1	1 *	0.2	0	0	0 *	0	0	0	0	0	1	4.0	0.1	0.1
Total	52,322	100	134,245	100	663	100	501	100	33	100	17	100	15	100	25	100	53,033	134,788

* Corrected standardized residuals lower than 1.96 in absolute value.

**Table 2:** Odds ratio age vs. severity.

Severity	Light		Serious		Very Serious		Fatal	
Category	Nurse	N.Assistants	Nurse	N.Assistants	Nurse	N.Assistants	Nurse	N.Assistants
Sex	O.R	O.R	O.R	O.R	O.R	O.R	O.R	O.R
Yong workers (18 to 45 years)	3.153	2.559	0.311	0.373	0.322	0.325	0.672	0.975
Senior workers (older than 45 years)	1	1	1	1	1	1	1	1
C.I	2.653–3747	2.117–3.093	0.260–0.372	0.305–0.455	0.145–0.715	0.106–0.997	0.239–1.888	0.455–2.137

**Table 3:** Contingency table Sex vs. Severity.

Severity	Light				Serious				Very Serious				Fatal				Total	
Category	Nurse		N.Assistants		Nurse		N.Assistants		Nurse		N.Assistants		Nurse		N.Assistants		Nurse TAR	N.Assistants TAR
Sex	Num	LAR	Num	LAR	Num	SAR	Num	SAR	Num	VSAR	Num	VSAR	Num	FAR	Num	FAR		
Men	6419	12.3	10,492	7.8	171	25.8	45 *	11.8	13	39.4	2 *	11.8	5	33.3	8	32.0	12.5	7.8
Women	45,903	87.7	123,753	92.2	492	74.2	456 *	88.2	20	60.6	15 *	88.2	10	66.7	17	68.0	87.5	92.2
Total	52,322	100	134,245	100	663	100	501	100	33	100	17	100	15	100	25	100	53,033	134,788

* Corrected standardized residuals lower than 1.96 in absolute value.

**Table 4:** Odds ratio sex of workers.

Severity	Light		Serious		Very Serious		Fatal	
Category	Nurse	N.Assistants	Nurse	N.Assistants	Nurse	N.Assistants	Nurse	N.Assistants
Sex	O.R	O.R	O.R	O.R	O.R	O.R	O.R	O.R
Men	0.752	0.386	1.163	2.480	1.571	4.574	5.547	3.515
Women	1	1	1	1	1	1	1	1
C.I	0.569–0.995	0.326–0.457	0.856–1.581	2.080–2.957	0.359–6.869	2.274–9.199	2.393–12.856	1.201–10.286

**Table 5:** Odds ratio length of service.

Severity	Light		Serious		Very Serious		Fatal	
Category	Nurse	N.Assistants	Nurse	N.Assistants	Nurse	N.Assistants	Nurse	N.Assistants
Sex	O.R	O.R	O.R	O.R	O.R	O.R	O.R	O.R
<5 years	0.947	1.230	1.033	0.800	1.706	1.011	0.948	0.973
>5 years	1	1	1	1	1	1	1	1
C.I	0.815–1.099	0.688–0.962	0.855–1.207	0.672–0.953	0.860–3.386	0.390–2.620	0.597- 2.663	0.444–2.134

## Data Availability

Not applicable.
